# Preclinical evaluation of FAP-targeted PET imaging to investigate CAF responses to radiotherapy

**DOI:** 10.1186/s40644-026-01010-2

**Published:** 2026-02-27

**Authors:** Kristin Lode, Sindhu Kancherla, Yngve Guttormsen, Rodrigo Berzaghi, Vera Susana Maia, Angel Moldes-Anaya, Turid Hellevik, Mathias Kranz, Inigo Martinez-Zubiaurre

**Affiliations:** 1 Department of Clinical Medicine, Molecular Inflammation & Irradiation Research Group, UiT the Arctic University of Norway, Tromsø, Norway; 2 Department of Clinical Medicine, Nuclear Medicine and Radiation Biology Research Group, UiT the Arctic University of Norway, Tromsø, Norway; 3 Department of Radiation Oncology, University Hospital of Northern Norway, Tromsø, Norway; 4 PET Imaging Center, University Hospital of Northern Norway, Tromsø, Norway; 5 Department of Medical Biology, Tumor Biology Research Group, UiT the Arctic University of Norway, Tromsø, Norway; 6 Department of Clinical Medicine, University of Tromsø, Tromsø, N-9037 Norway

## Abstract

**Background:**

Cancer-associated fibroblasts (CAFs) are influential elements of the tumor microenvironment with significant roles in tumor progression and therapy resistance. However, the effects that radiotherapy has on CAFs dynamics remain understudied. This study aimed to provide a non-invasive PET imaging approach to quantify radiation-induced changes in cancer-associated fibroblasts in preclinical tumor models.

**Methods:**

The FAP-specific radiotracer [^18^F]AlF-FAPI-74 was used to monitor CAFs dynamics following precision external beam radiotherapy (RT) in two syngeneic subcutaneous murine tumor models (LLC and CT26). Tumors were irradiated via two radiation regimens (1 × 12 Gy or 2 × 6 Gy), and dynamic PET/MR imaging was performed 7 days after RT. Additionally, the dynamics of FAP+ CAFs in tumors were quantified ex vivo via flow cytometry and immunohistochemistry.

**Results:**

Tumor-targeted irradiation led to a significant reduction in tumor size. Uptake of [^18^F]AlF-FAPI-74 in subcutaneous tumors was low but significantly above muscle-background values. Quantification of standardized uptake values (SUVs) from static PET images revealed a twofold increase in the PET signal in LLC tumors irradiated with two fractions of medium-dose RT (2 × 6 Gy). Ex vivo analysis confirmed the low abundance of FAP+ cells in tumors and demonstrated similar RT-induced changes in CAF levels across the different models.

**Conclusions:**

Our findings suggest that CAFs constitute a relatively small cell population in subcutaneously transplanted tumor models, and that fractionated radiotherapy may induce a moderate increase in FAP^+^ cells in LLC tumors. Additionally, we demonstrated that [^18^F]AlF-FAPI-74 is a reliable biomarker for evaluating the number of FAP+ stromal cells in tumors and for addressing potential therapy-induced changes in CAFs.

**Supplementary Information:**

The online version contains supplementary material available at 10.1186/s40644-026-01010-2.

## Background

Among all the stromal cells that reside in the tumor microenvironment (TME), cancer-associated fibroblasts (CAFs) are among the most abundant and critical components, providing not only physical support for tumor cells but also key roles in promoting or restraining tumorigenesis in a context-dependent manner [1]. The presence of CAFs in the TME is frequently correlated with increased angiogenesis, invasion and metastasis and is thus associated with a poor prognosis in a wide variety of solid malignancies [2]. In addition, CAFs are recognized as mediators of immunosuppression in the TME [3, 4]. Notably, recent reports highlight the participation of CAFs in therapy resistance [5, 6]. In the context of radiotherapy (RT), the ultimate role of CAFs in therapeutic outcomes remains unresolved [7]. While some studies claim that ionizing radiation (IR) has detrimental effects on CAFs by inducing growth arrest and impaired motility [8, 9], others argue that exposing fibroblasts to radiation promotes their conversion into a more activated and aggressive phenotype [10]. Hence, further research is needed to increase our understanding of CAF responses to ionizing radiation and to elucidate the potential role that CAFs may play in tumor radioresistance.

Given its important role in cancer progression and therapy resistance, the tumor stroma represents an attractive target for delivering diagnostic and therapeutic compounds [11]. Several approaches have been applied to target CAFs with novel radiolabeled probes based on antibodies, peptides and small molecule inhibitors in different cancer types [12]. Currently, some of the most commonly used strategies involve radiotracers that target fibroblast activation protein (FAP) [13]. FAP is a membrane-bound proline-specific serine protease with dipeptidyl peptidase and endopeptidase activities [14] that is known to degrade denatured type-I collagen, alpha-2 antiplasmin and FGF21 in vivo [15]. High FAP expression is associated with pathologic remodeling of the extracellular matrix, a process inherent to the development of solid malignancies [16]. Reactive stromal fibroblasts, i.e., CAFs in solid tumors, are characterized by abundant surface expression of FAP, and their presence is frequently associated with poor prognosis, whereas low or no expression is observed in normal fibroblasts in healthy tissues in humans [14]. However, FAP can also be expressed on stromal fibroblasts during nonmalignant processes, such as tissue remodeling, fibrosis, wound healing and inflammation [17].

The development of the selective FAP inhibitor UAMC-1110 has led to promising radiolabeled FAP inhibitors (FAPIs) that have been tested in different tumor entities [18, 19]. Quinoline-based FAP inhibitors specifically bind to the enzymatic domain of FAP prior to cellular internalization. Different methods for the conjugation of quinoline-based FAP ligands with chelators suitable for radiolabeling have been developed [18, 20]. In this study, we used a FAPI-74 variant that includes a NOTA chelator. Other FAPI-based radiotracers have been successfully used as tumor-specific imaging biomarkers in preclinical and clinical models [21–23]. Here, we investigated the impact of radiotherapy on CAFs in vivo via PET/MR imaging of a FAP-targeting radiotracer in two different preclinical tumor models. The results indicate low but significant tumor uptake of FAPI-74 and some background PET signals in joints. Fractionated medium-dose radiotherapy induces twofold enhanced tracer uptake, which is visible as hyperintense tumor-specific PET signals.

## Materials and methods

### Cell cultures

Murine cell lines of Lewis Lung Carcinoma (LLC) expressing luciferase (LL/2-Luc2) and colon carcinoma (CT26) were purchased from ATCC (Virginia, USA; Cat # CRL-1642-LUC2 and # CRL-2638). In this study, the LL/2-Luc2 cell line is referred to as LLC for simplification. LLC cells were cultured in high-glucose RPMI (Sigma Life Science; Cat #D5796) supplemented with 10% FBS, 100 U/mL penicillin, 100 μg/mL streptomycin and 2 μg/mL blasticidine, whereas CT26 cells were cultured in RPMI-1640 (Sigma Life Science; Cat # R8758) supplemented with 10% FBS, 100% U/mL penicillin and 100 μg/mL streptomycin.

### In vivo models

Female C57BL/6J and BALB/cJ mice (aged 6–8 weeks; Charles River, Sulzfeld, Germany) were acclimatized in the local animal facility for a minimum of five days prior to experimentation. The animals had access to water and standard chow (Scanbur, BK, Norway) *ad libitum*. All procedures and experiments involving animals were conducted according to regulations of the Federation of European Laboratory Animal Science Association (FELASA) and approved by the Norwegian Food and Safety Authority (FOTS ID 18,956 and 25,795). LLC, which is syngeneic to C57BL/6J mice, and CT26, which is syngeneic to BALB/c mice, were used in this study. Prior to inoculation, all the cell lines were tested and proven pathogen-free by Idexx Bioanalytics (Mouse Comprehensive Test). For inoculation, the cells were prepared in sterile RPMI and Geltrex^TM^ (Gibco, Cat # A1413201) at a 1:1 ratio. For tumor cell inoculation, 100 μL of the suspension (5x10^5^ cells) was injected subcutaneously into the right hind flank of each mouse under anesthesia. During growth, tumors were measured at 3X/week via a digital caliper, and volumes were calculated via the modified ellipsoidal formula ($V = {1 \over 2}\left( {length \times widt{h^2}} \right)$).

### Animal tumor irradiation

The animals were subjected to image-guided radiotherapy in a dedicated small-animal irradiator (Precision X-ray, North Branford) when the tumors reached 5–6 mm in diameter (8–10 days after cell inoculation). The animals were anesthetized prior to CT imaging and RT delivery via continuous isoflurane gas (0.5 L/min oxygen with 4% isoflurane) in the induction chambers. Anesthesia was maintained throughout the entire procedure via continuous isoflurane gas via a nose cone (0.4 L/min oxygen with 2% isoflurane).

Anatomical CT images were acquired for each tumor and imported into the SmART-plan advanced treatment planning system for tumor delineation, pretreatment dose calculations and plan evaluations, as previously described [24]. Next, RT was delivered to the tumors via the treatment plan, which used two opposing photon beams, with a maximum energy of 225 kV, a dose rate of 3.1 Gy/min, and a collimator size of Ø=10 mm. The radiation regimens included a single high dose of 12 Gy (1x12 Gy) or two intermediate doses of 6 Gy (2x6 Gy) 24 hrs apart.

### Radiosynthesis of [^18^F]AlF-FAPI-74

Chemicals for radiotracer production were purchased from VWR (Oslo, Norway), unless otherwise stated. Fluoride (^18^F-) batches (~10 GBq) were locally produced via the nuclear reaction 18O(p,n)^18^F in the “PETtrace 860” medical cyclotron (GE Healthcare, Uppsala, Sweden), which uses a proton beam (16.5 MeV) to bombard a niobium target prefilled with [^18^O]H_2_O (Rotem Industries, Israel).

Radiosynthesis of [^18^F]AlF-FAPI-74 was performed following the procedure of Dahl [25] and Giesel [21] et al., with some modifications. Analysis of the formulated product [^18^F]AlF-FAPI-74, the nonradioactive standard [^19^F]AlF-FAPI-74 and the precursor FAPI-74 were performed on an X-Bridge BEH C18 (4.6X150 mm, 3.5 μm) (Waters, Oslo, Norway) analytical column by gradient elution with a mobile phase (0.1% NH3 and 95% acetonitrile) for 30 min at a flow rate of 1 mL/min.

### In vitro and in vivo [^18^F]AlF-FAPI-74 metabolic stability tests

The in vitro metabolic stability of [^18^F]FAPI-74 was first studied in mouse plasma obtained by full-blood centrifugation (2500 rpm, 5 min, 4°C). Next, plasma was incubated with ~10 MBq of [^18^F]AlF-FAPI-74 in a shaking water bath (37°C) with circulation, and samples (100 µL) collected 5, 15, 30, 45, 60 and 120 min after adding the radiotracer, followed by inclusion of 100 µL ice-cold acetonitrile per sample to deproteinize the plasma. Upon a reaction-time for indicated timepoints, samples were subjected to vortex mixing followed by ultrasound bath (5 min) and final centrifugation (4 °C, 10 min, 14,500 rpm). A volume of 100 uL from the clear supernatants were analyzed by HPLC, following the procedure described above (Fig S1b-c).

For checking the in vivo stability of [^18^F]AlF-FAPI-74, sample preparations were carried out in a similar manner as for the in vitro stability study, except that animals (*n* = 3 per timepoint per strain) were injected i.v. with 100 ± 20 MBq [^18^F]AlF-FAPI-74 (in 50 μ L) through a tail-vein catheter, blood collected by cardiac puncture (1 mL) at different time points (15, 30 and 60 min) post-injection, with further sample processing as described for the in vitro stability test (Fig S1d-e)

### In vivo imaging procedures of whole-body PET and MRI

Animals were anesthetized with isoflurane (4% induction in O_2_) and subjected to simultaneous whole-body PET and MRI (MR Solutions 7.0T PET/MR, Guildford, UK) one day prior to the first dose of RT (baseline pre-RT FAPI values) and one week post-RT. Since the uptake of 18F-FAPI-74 in tumors at early stage of development was very low (in line with background signal in muscle), the results have not been included in the main figures of the study. For PET/MR imaging, 7 MBq (±3 MBq) in 100 μL [^18^F]AlF-FAPI-74 was injected intravenously via a retroorbital route under anesthesia (isoflurane, 0.4 L/min, 2% in O_2_). Radioligand biodistribution was compared between the two routes of administration, revealing nearly identical biodistribution patterns (not shown), which has also been demonstrated by others [26]. Following tracer injection, the animals were returned to their cages and remained awake until imaging, 40 min post-injection. During (PET/MR) imaging, the animals were placed in the prone position in a multimouse bed (Mouse hotel, MINERVE, Esternay, France) with two mice. To acquire static images, PET scans were performed for 20 min in all the animals. The lung adenocarcinoma (LL/2-Luc2) and colon carcinoma (CT26) tumor bearing animals were subjected to 60 minutes dynamic PET/MRI scanning starting simultaneously with the radiotracer injection The list-mode data were rebinned into 24x5s, 8x60s, and 10x300s time frames and reconstructed into 3D datasets via OSEM (two iterations, 32 subsets) and DICOM data exported for further analysis.

### PET data analyses (static and dynamic imaging)

In vivo organ biodistribution was assessed and quantified on PET/MR images (dynamic imaging). For each animal, 3D organs were delineated on T1-weighted MR images, followed by PET image co-registration. The muscle volume of interest (VOI) was defined as the volume around the whole thigh muscle of the mouse hindleg, excluding the bone (hypointense/dark in MRI). The average activities (in kBq/mL) were extracted from all the organ VOIs and converted into standardized uptake values (SUVs) or percentages of the injected dose/mL (%ID/mL), using the decay value corrected for the total injected dose, which is equivalent to the percentage injected dose per gram (%ID/g) and was calculated for in vivo organ biodistributions [17]. The dynamic PET imaging data was visualized in time-activity curves to analyze the pharmacokinetic profile of [18F]AlF-FAPI-74 over time.

The tumor volumes were segmented via PMOD (v4.3, PMOD Technologies LLC, Switzerland) via T1-weighted MRI, and the SUV or %ID/g for the VOIs were obtained. For tumors, regions with greater accumulation of [^18^F]AlF-FAPI-74 were defined on the basis of PET data using a threshold of 75% of the maximum VOI value to obtain the biologically active tumor volume (BTV). BTV75 was defined by retaining the tumor subvolume comprising the top 25% of PET signal intensities, i.e., voxels within the tumor VOI with uptake ≥75% of the maximum SUV. This region was used to highlight areas with the highest FAPI tracer accumulation and to evaluate radiotherapy-induced changes in biologically active tumor regions. The muscle VOI in the contralateral leg from the tumor was used to obtain SUV values for physiological perfusion of [^18^F]AlF-FAPI-74 to calculate the tumor-to-background ratio. The resulting quantitative data are presented as “fold-change uptake” in the tumor relative to the contralateral muscle.

### Ex vivo tumor analyses of CAFs

For immunohistochemistry evaluations, tumors were excised 7 days post-RT, fixed in paraformaldehyde (4% in PBS) immediately after resection and embedded in paraffin blocks. A Discovery Ultra Research instrument (Roche 05987750001) was used for automated preparations and immunohistochemical staining of murine tumor tissue sections (4 μm thick). An anti-mouse αSMA antibody (D4K9N, Cell Signaling) for identifying and quantifying CAF levels in tumors was used at a 1:100 dilution for staining. The antibody was validated for IHC-P (formalin-fixed and paraffin-embedded murine tissue) by the supplier. The dilutions, incubation times, antigen retrieval methods and temperatures were optimized in-house. Staining and antibody specificity were verified by an internal tissue control containing several normal and cancer tissues. Negative controls were generated by omitting the primary antibody from the staining procedure. Positive staining of the samples was quantified digitally via QuPath software for image analysis (version 0.0.5.1, tool: Positive Cell Detection).

In addition, the presence of FAP^+^ cells in irradiated versus nonirradiated tumors was determined by flow cytometry. For this purpose, tumors were also collected 7 days post-RT, minced, and enzymatically digested, and the resulting cell suspensions were stained with viability dye and a murine anti-FAP antibody (Bioss Antibodies, Cat# bs 5758-A680). Data was obtained by flow cytometry from cells gated according to their scatter properties (FSC-A vs SSC-A) and doublet exclusion (FSC-A vs FSC-H). Cell debris was excluded from the analyses on the basis of scattered signals. The data acquired via flow cytometry were analyzed via FlowJo software (Version v0.10.10; TreeStar, OR, USA).

### Statistical analyses

All values are expressed as the mean ± standard deviation (SD). Statistical analyses were performed via GraphPad Prism (GraphPad Software Inc., La Jolla, CA). Comparisons of data from three or more experimental groups were conducted via one-way ANOVA followed by Dunnett post hoc corrections for multiple comparisons providing that the conditions of normally distributed residuals were met. When residuals were not normally distributed, comparisons of three or more groups were conducted via nonparametric Kruskal-Wallis with Dunn’s post hoc test for multiple comparisons. Comparisons of data between two groups were conducted via unpaired two-tailed Student’s t tests. The level of significance was defined as *p* ≤ 0.05. Six animals (*n* = 6) were included per experimental group.

## Results

### Radiotherapy effects on tumor growth kinetics

**Fig. 1:**
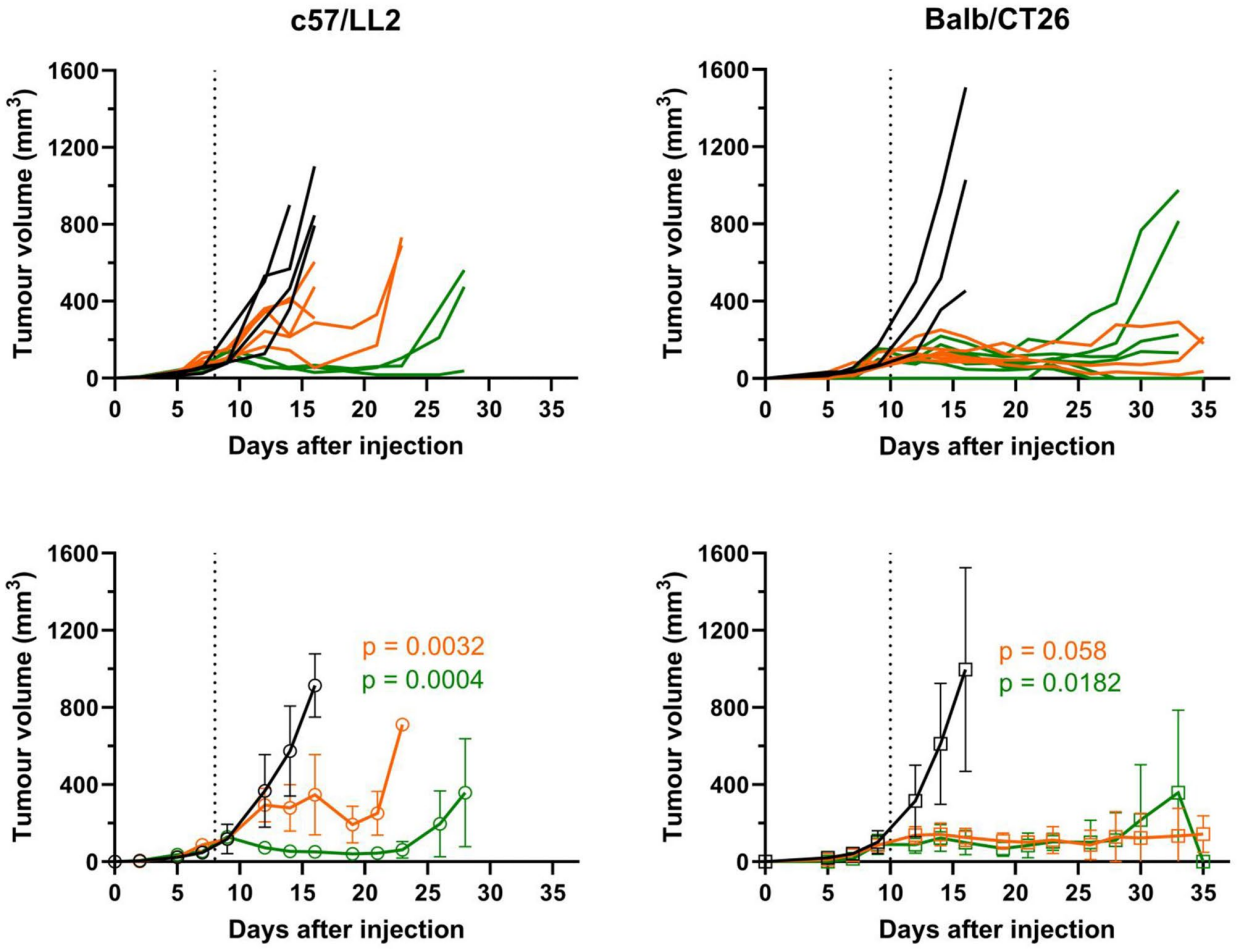
Tumor growth responses to radiotherapy. The effect of focused RT on tumor growth was evaluated over time. In the top panels, individual lines represent tumor growth from single mice, whereas curves in the bottom panels represent the mean values for each experimental group ± SDs (*n* = 6). The black vertical dotted line in the figures indicates the timepoint for CT-guided RT to tumors, i.e., day 8 or 10 after tumor cell injection in C57BL/6J and BALB/c mice, respectively. Statistics: *p*-values are based on non-parametric Kruskal-Wallis test with Dunn’s correction for multiple comparisons. The treatment effect was evaluated at day 16 post cell implantation. This reflects the latest time points of the control groups of both models and allow the neglection of time as a parameter of influence in tumor volume

The effects of radiotherapy on tumor growth were analyzed in two different tumor models. Tumor irradiation was performed during exponential growth once the tumors reached 5–6 mm in diameter. Both tumor models exhibited similar tumor growth kinetics in the nonirradiated groups; however, compared with LLC tumors, CT26 tumors presented greater variation in growth, as indicated by a greater SD (Fig. 1, bottom panels). In the LLC model, both radiation regimens (2x6 Gy and 1 × 12 Gy) induced significant delays in tumor growth, although the effects were more pronounced in the 1 × 12 Gy group (Fig. 1, left panels). In the CT26 model, both radiation regimens induced potent and durable growth delays in a comparable way (Fig. 1, right panels), with some animals in the 2 × 6 Gy treated group showing complete tumor regression.

### [^18^F]AlF-FAPI-74 uptake in tumor lesions

**Fig. 2:**
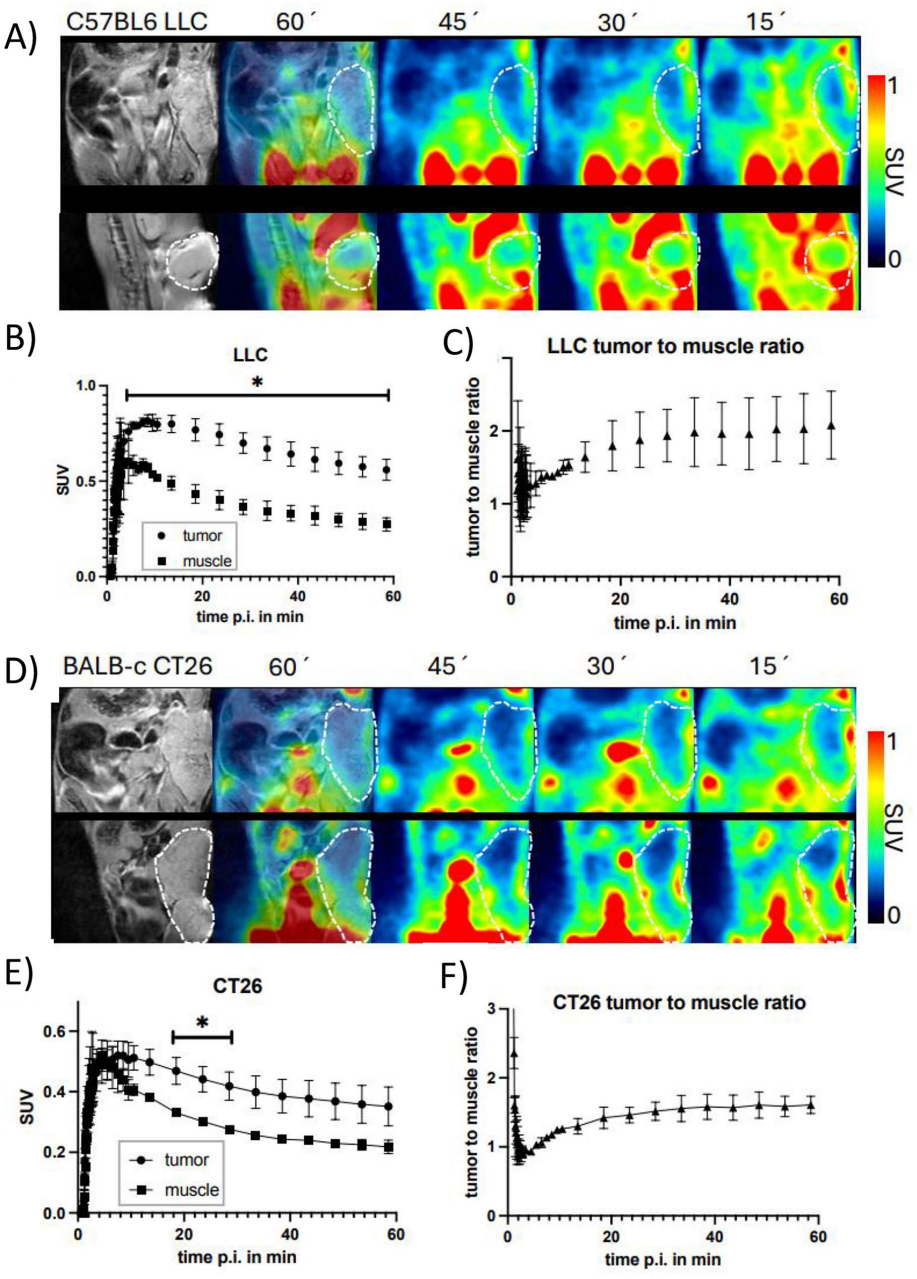
Dynamic uptake of [^18^F]-AIF-FAPI-74 (SUVmean) in subcutaneously implanted lung adenocarcinoma (LL/2-Luc2) and colon carcinoma (CT26) tumors. Time‒activity curves (SUVmean) and tumor-to-background ratios of the radiotracer in syngeneic murine tumor models over a 60-minute dynamic PET imaging period. (**A–C**) Data from Lewis lung carcinoma (LLC) tumor-bearing C57BL/6 mice (*n* = 2) showing radiotracer uptake in tumor and muscle tissue (**A**), along with corresponding tumor-to-muscle, tumor-to-blood, and tumor-to-lung ratios (**B–C**), Illustrating favorable tumor-to-background contrast over time. (**D–F**) Similarly, in CT26 colon carcinoma-bearing BALB/c mice (*n* = 2), radiotracer uptake is shown in tumors and muscle (**D**), with associated tumor-to-muscle and tumor-to-blood ratios plotted over time (**E–F**). Rapid tracer accumulation within tumors was observed in both models, with retention plateauing at later time points, indicating stable intratumoral localization. The tumor-to-organ ratios progressively increase, suggesting ongoing clearance from nontarget tissues and improved imaging contrast. Statistical analyses followed a two-tailed t-tests comparisons

To assess tumor-associated [^18^F]AlF-FAPI-74 uptake, tumor volumes were carefully delineated in a series of MR images and coregistered with the corresponding PET images (Fig. 2A&D). The injected dose was decay-corrected to the starting time for PET scanning, and both the SUVmean (Fig. 2B&E) and the SUV max (supplemental material, Fig S2) values were calculated. To compensate for physiological tissue perfusion, a VOI in the contralateral leg muscle was delineated and used as a reference region in each animal to calculate the T/M ratio (Fig. 2C&F). Furthermore, tumor-to-blood ratios and tumor-to-lung ratios were calculated to observe blood clearance in the LLC model (Fig. S2) and the CT26 model (Fig. S3). In general, tumor uptake was low and heterogeneous in both the LLC and the CT26 models but had values higher than the muscle background (Fig. 2E). Notably, the maximum uptake of [^18^F]AlF-FAPI-74 was observed in the peripheral tumor regions, where it decreased toward the core of the tumor (Fig. 2A&D, and Fig. S3).

### Radiotherapy-induced changes in [^18^F]AlF-FAPI-74 tumor uptake

**Fig. 3:**
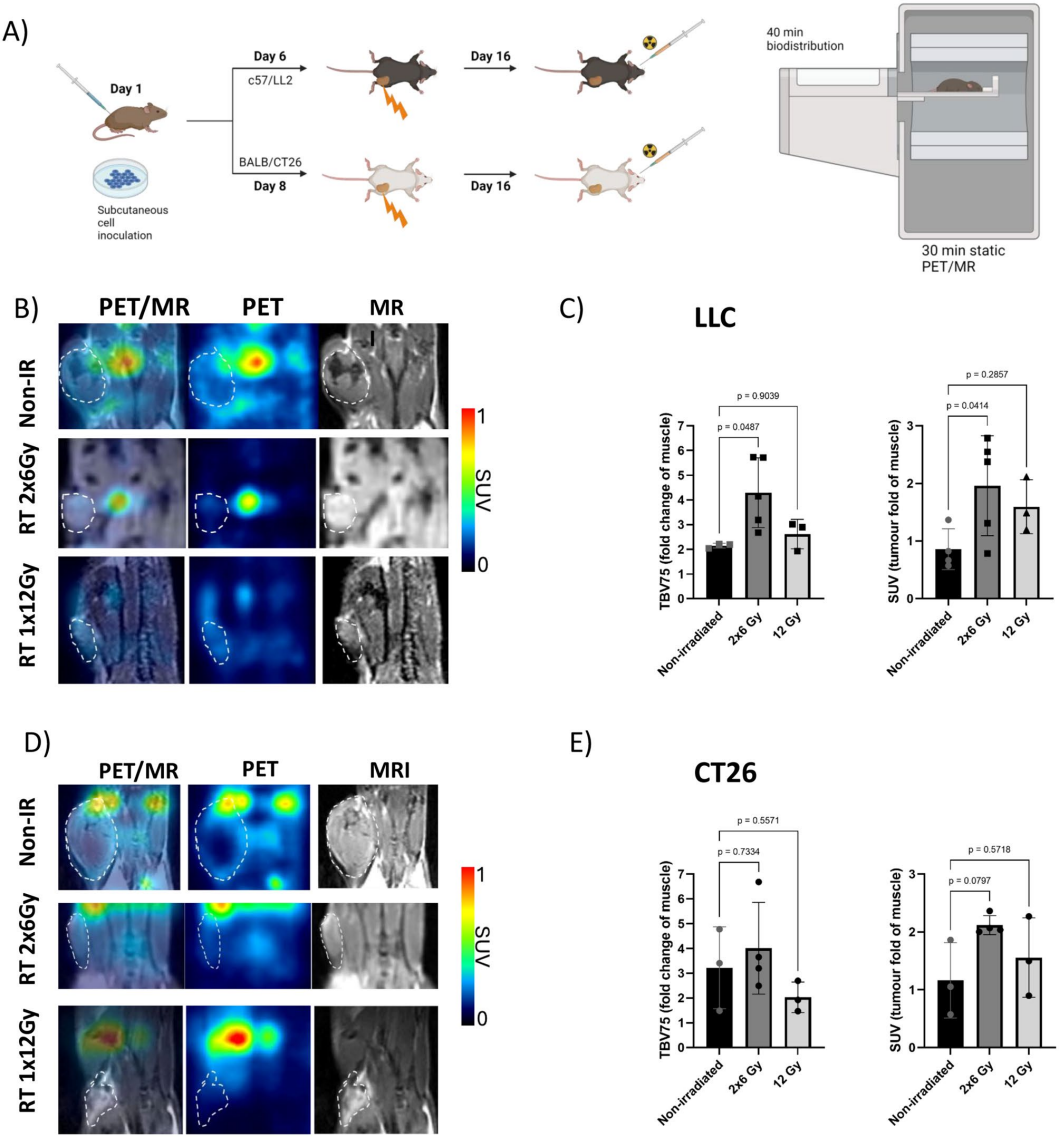
Changes in tumor-specific [^18^F]-AIF-FAPI-74 uptake following radiotherapy. (**A**) Schematic overview of experimental timeline. Tumor uptake of [^18^F]AlF-FAPI-74 in irradiated and nonirradiated LLC (**B**) and CT26 (**C**) Tumors. Tumor-specific uptake was quantified on the basis of tumor delineations on MR images prior to PET coregistration, and tracer uptake in the contralateral muscle was used as a reference. Tumor-specific uptake was normalized to the background signal in the contralateral muscle (right panels). The values are average values of the VOI of the whole tumor as the SUV, whereas BTV75 represents the “biological-tumor-value-75” of the highest 75th percentile tracer accumulation in the tumor VOI. The bars represent the mean values ± SD from six animals. Six animals (*n* = 6) were included per experimental group. Statistics: one-way ANOVA followed by Dunnett post hoc corrections for multiple comparisons

When comparing the tumor uptake of [^18^F]AlF-FAPI-74 in untreated animals, LLC tumors displayed the lowest absolute tumor uptake and tumor-to-muscle ration compared to the CT26 model (Fig. 3B&D). Interestingly, in this model, the fractionated radiation regimen (2x6 Gy) increased the overall SUV twofold, reaching statistically significant differences (*p* = 0.04) compared with nonirradiated controls. However, only minor variations in the PET signal were observed when untreated animals were compared with those in the single-high-dose (1x12 Gy)-treated group (*p* = 0.9) (Fig. 3B). When BTV75 was used as an alternative quantitative approach, the outcomes showed similar trends, with a twofold increase in the signal in the 2 × 6 Gy group, which also reached statistical significance (*p* = 0.04).

Similar effects of radiation were observed in the BALB/c CT26 model (Fig. 3D). However, in this model, the differences between the untreated group and the fractionated radiation group (2x6 Gy) did not reach statistical significance for either the SUV_mean_ (*p* = 0.079) or the relative BTV75 value (*p* = 0.73) (Fig. 3D). Interestingly, the BTV75 values in the 1 × 12 Gy radiation group were lower than those in the untreated group (Fig. 3D). In both models, the fractionated regimen (2x6 Gy) resulted in the highest tumor-specific BTV75 PET signal relative to muscle, although statistical significance was reached only in the LLC model.

When the spatial distribution of [^18^F]AlF-FAPI-74 in tumors was examined, heterogeneous patterns were observed, irrespective of the tumor model used. The maximum intensity PET signal (SUVmax) was typically observed at the periphery of tumors, with limited accumulation in the central areas (Fig. 3A&C and Fig S3). This trend was observed in both the tumor models and all the treatment groups. Heterogenous tracer uptake was most evident in the larger, nonirradiated tumors, where the PET signal was clearly visible as a ring around the tumor, as shown in Fig. 3A&C and in Fig. S3.

### Ex vivo analyses of intratumoral CAFs

**Fig. 4:**
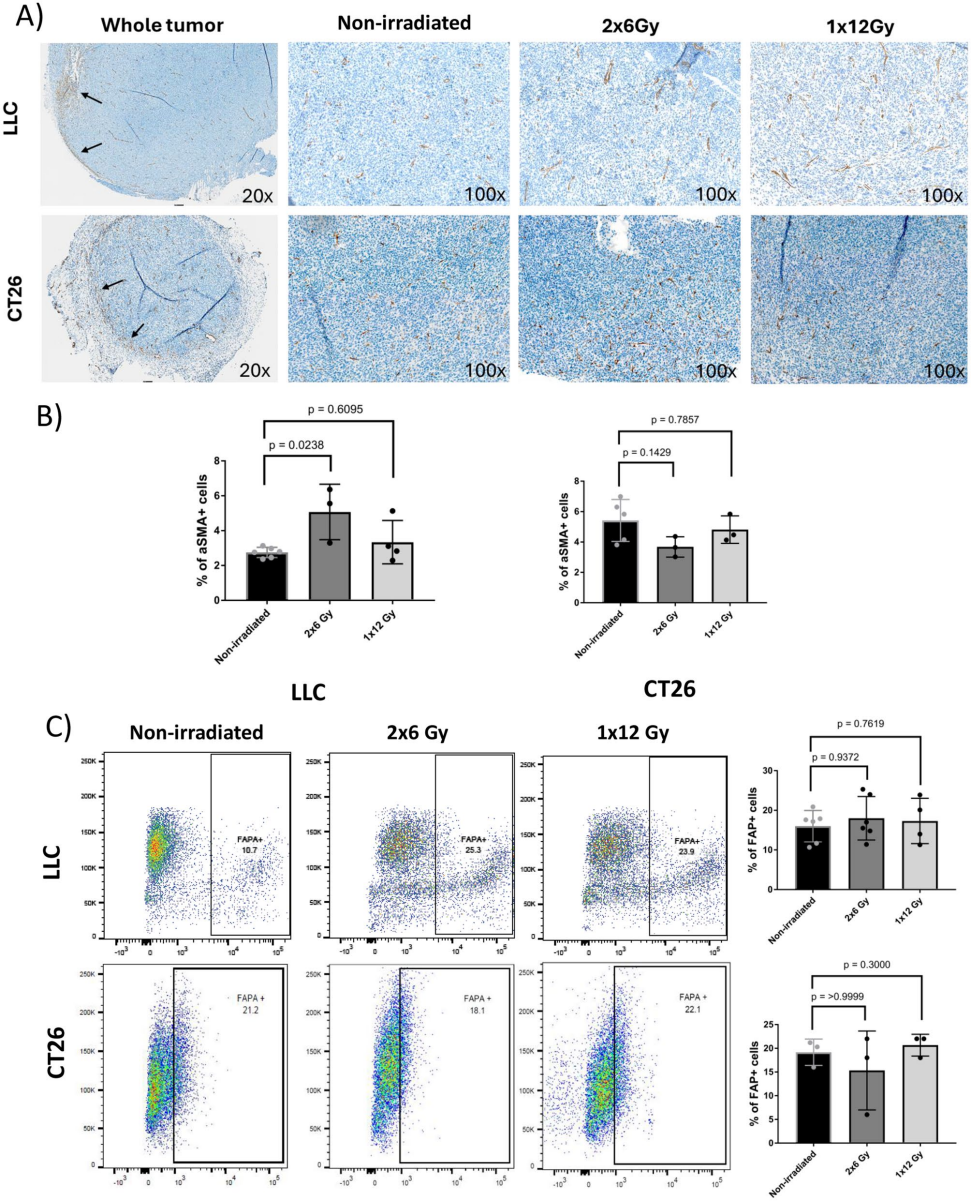
Ex vivo analyses of tumor-infiltrating CAFs by flow cytometry and immunohistochemistry. **A**) Immunohistochemistry micrographs of excised LLC and CT26 tumors, showing intratumoral αSMA+ CAFs (brown color) from tumor samples retrieved 1 week post-RT. The arrows indicate areas in the tumor periphery with enhanced aSMA+ CAF staining. **B**) Digitally quantified +αSMA expression on whole tumor tissue slides (*n* = 6 per strain). **C**) Flow cytometry analyses to quantify FAP+ cells in enzymatically digested fresh tumor tissue samples retrieved 1 week post-RT. The bars represent the mean values ± SDs. Sham-irradiated tumors (non-irradiated) and tumors treated with 2x6Gy and 1 × 12 Gy were compared (*n* = 6). Statistics: one-way ANOVA followed by Dunnett post hoc corrections for multiple comparisons

To validate findings via quantitative PET imaging, the content of intratumoral CAFs in tumor tissue specimens was also analyzed via immunohistochemistry (IHC) and flow cytometry. IHC analyses revealed that LLC tumors presented very poor stroma development and low CAF infiltration, as illustrated by limited infiltration of αSMA^+^ cells (brown signal in micrographs, Fig. 4A) and negligible expression of extracellular matrix proteins (analyzed by Masson´s trichrome, not shown). Computer-assisted quantification of αSMA expression revealed comparable levels between untreated and 1x12Gy-irradiated tumors (2.74% and 3.34% positive cells from the total number of cells, respectively) but significantly increased levels in the (2x6 Gy)-treated group (5.07% positive cells, *p* = 0.02) (Fig. 4A& B). In the CT26 model, tumor irradiation did not affect the level of αSMA expression (Fig. 4A& B).

Moreover, the percentage of FAP^+^ cells in the total fraction of viable cells extracted from nonirradiated or irradiated tumors was also determined via flow cytometry (Fig. 4C). Among the fraction of viable cells in nonirradiated LLC tumors (from C57BL/6J mice), nearly 40% were FAP+. Notably, the fraction of FAP+ cells increased to 55% in the 2 × 6 Gy irradiation group, which reached statistical significance, whereas the FAP+ values for the 1 × 12 Gy group remained similar to those of the untreated group (47%). Compared with those in LLC lung tumors, colon carcinoma CT26 tumors in BALB/cJ mice presented a lower proportion of FAP^+^ cells in the viable population (~20%), but no changes in FAP+ cells were observed between the experimental groups (Fig. 4C).

## Discussion

In this study, the main aim was to explore the effects of radiation on tumor fibroblasts and to evaluate PET imaging with the radiotracer [^18^F]AlF-FAPI-74 as a biomarker to study the dynamics of FAP+ stromal cells in the context of cancer therapy. With the intention of applying a broad approach, we reproduced the results in two different syngeneic murine tumor models: the Lewis lung adenocarcinoma model LLC and the colon carcinoma model CT26. With respect to radiotherapy, we used two different regimens: a single-high dose (1x12 Gy) and a fractionated medium-dose regimen (2x6 Gy). These results indicate that focused external beam radiotherapy, especially when given at fractionated (medium–high) doses, may induce a moderate increase in intratumoral FAP+ cells. Moreover, this radiation-induced increase in FAP+ expression was more prominent in the LLC/C57BL/6 lung carcinoma model than in the CT26/BALB/c colon carcinoma model. Additionally, we demonstrated that FAPI-74 is a reliable biomarker for evaluating the number of FAP+ stromal cells in tumors and addressing potential therapy-induced changes in CAFs.

In this work, we used the FAPI-74 variant for PET imaging. Our choice was based on several practical and technical advantages that FAPI-74 offers over other FAPI variants, particularly in clinical and research settings. FAPI-74 is compatible with both ^68^Ga and ^18^F labeling, offering flexibility in tracer production. The ^18^F-labeled FAPI-74 variant, in particular, benefits from a longer half-life (110 minutes vs. 68 minutes) and thus better clinical applicability. Additionally, [^18^F]AlF-FAPI-74 has demonstrated lower positron energy than ^68^Ga-labeled variants do, resulting in better spatial resolution and image quality [27]. Recent studies have shown that ^18^F-FAPI-74 PET/CT provides high diagnostic accuracy, particularly in detecting metastatic lymph nodes and small lesions, which may be underestimated with other tracers due to partial volume effects. Its high tumor-to-background ratio and rapid clearance from nontarget tissues make it suitable for a wide range of oncologic applications [28]. The radiolabeled product [^18^F]AlF-FAPI-74 was synthesized with a radioactivity yield of 15% and a radiochemical purity of > 99%. Extensive in vitro (plasma) and in vivo stability tests confirmed satisfactory isotope retention in the chelator and no metabolization of the compound within the experimental timeframe (~1 h) (Fig. S1). These results support the notion that the PET signal from static scans acquired 1 h after [^18^F]AlF-FAPI-74 injection corresponds to images derived from the intact tracer.

Several PET radiopharmaceuticals with common binding motifs against FAP (FAP inhibitors) have been tested in humans, with promising results [29]. One of the first developed FAPI compounds, [^68^Ga]Ga-FAPI-04, was successfully tested as an imaging agent in 28 different cancer types in humans; this agent displays limited background signals and provides high image contrast in tumors [30]. In our study, the NOTA chelator-conjugated FAPI-74 was synthesized and first tested in humans by Giesel et al. [21]. In this landmark study, high-contrast images of primary tumors, lymph nodes and distant metastases were obtained 1 h after injection, which could support target-volume-definition for guiding radiotherapy delivery. Importantly, no uptake exceeding the perfusion-dependent background was observed in major organs, including intestines and bone structures.

In tumor-bearing animals, [^18^F]AlF-FAPI-74 uptake in tumors was slightly greater than uptake in muscle, and most of the radiotracer accumulated in the periphery of the tumor. These data are consistent with poorly developed stroma in subcutaneously transplanted tumors, characterized by nearly undetectable extracellular matrix deposition and a low abundance of FAP-expressing fibroblasts [9, 31]. Our results align well with observations in other preclinical models using tumor cells not genetically modified to overexpress FAP, where the only source of FAP arises from endogenously recruited levels of tumor-activated fibroblasts [19]. In a recent study by Liu M et al., the authors investigated radiation-induced changes in FAPI tumor uptake using the [^18^F]AlF-NOTA-FAPI-04 variant along with subcutaneously transplanted LLC cells as a model [32]. Similar to our observations, the authors observed a weak FAPI-04 signal in tumors compared with FDG, but in contrast, they reported a reduction in FAP expression and the FAPI-04 signal in tumors exposed to 1 × 15 Gy irradiation. In our study, an enhanced FAP signal was observed only after 2 × 6 Gy treatment, indicating that the response of FAP to radiation may markedly depend on the radiation regimens used in the experiments.

The effects of ionizing radiation on CAFs have been previously investigated in animal models; however, in most studies, the tumorigenic effects of irradiated CAFs have been studied after the coimplantation of (in vitro) irradiated CAFs with tumor cells [9, 33–35]. In fact, studies demonstrating the direct effects of radiotherapy on CAFs in situ are very rare [36, 37]. Hence, this study is one of the first to investigate tumor fibroblast dynamics following local external beam radiotherapy. Our data revealed a low abundance of αSMA+ CAFs in the stroma of subcutaneously transplanted LLC and CT26 tumors. Ex vivo analyses of resected tumors indicate that external radiotherapy may induce a moderate increase in CAF quantity (i.e., accumulation of FAP+ CAFs) in tumors but only by radiation applied in a fractionated manner with medium‒high radiation doses. In vivo image analyses using [^18^F]AlF-FAPI-74 revealed similar trends, with statistically significant differences only in the (2x6 Gy) RT group and only in the LLC/C57Bl6J model. The reasons for the observed differences between models are not known and difficult to predict since experiments were performed in two different tumor models in two different mouse strains. There could be multiple reasons for such variability. The baseline levels and activity of CAFs may differ between the LLC and the CT26 models. Additionally, the LLC and CT26 models represent different cancer types with distinct genetic and molecular profiles. These differences could affect how the tumor cells and stromal components, including CAFs, respond to radiation. Also, the intrinsic radiosensitivity of the tumor cells and stromal cells (including CAFs) may differ between the two models, leading to varying outcomes. Regarding radiation dose and fractionation, the 2x6Gy regimen involves two smaller doses of radiation delivered over time, while the 1x12Gy regimen delivers a single high dose. Fractionated radiation (2x6Gy) may allow for better targeting of CAFs by inducing sublethal damage and promoting reoxygenation, which can enhance the effects of subsequent doses. In contrast, a single high dose (1x12Gy) may not have the same impact on CAF dynamics. On the other hand, CAFs may respond differently to different RT regimens. Fractionated doses might induce a more pronounced effect on CAF activation, proliferation, or survival in the LLC model, while the 1x12Gy regimen is more efficient in inducing cellular senescence. In line with our observations in preclinical models, Verset et al. [38] reported higher αSMA/tumor epithelial area ratios after neoadjuvant radio(chemo)therapy in rectal cancer samples from patients, indicating that radiotherapy, when applied in specific regimens, may increase the quantity of CAFs in tumor lesions. An alternative explanation for this observation is that the surviving fraction of CAFs after treatment is greater than the surviving tumor epithelial fraction is, thus resulting in elevated CAF/tumor cell ratios but without affecting CAF infiltration or proliferation in tumors. Alternatively, our finding of twofold increases in FAP levels could be a consequence of the radiation-induced increase in the number of FAP surface receptors on CAFs. However, in our laboratory, we demonstrated that cell cultures of in vitro-irradiated CAFs from human NSCLC tumors do not respond to elevated FAP surface expression upon IR exposure (3x6 Gy or 1 × 18 Gy) (unpublished observations).

A clear limitation of our study is related to the use of subcutaneously transplanted tumor models, as these models do not closely recapitulate tumor-host tissue interactions, similar to endogenously formed tumors. In the former models, the tumor stroma is poorly developed, and consequently, the number of FAP-expressing cells, normally fibroblasts, is very low. This setback does not improve much when orthotopically transplanted tumors are used, since even after the transplantation of tumor cell lines in the organ of origin, the high proliferative rate of neoplastic tumor cells impedes the concomitant formation of proper tumor stroma [39]. In preclinical settings, the majority of published studies on FAP-targeting PET radiotracers use genetically engineered tumor cells overexpressing FAP [40, 41], which are artificial models that do not represent *bona fide* FAP expression in tumor lesions. Alternative animal models that more faithfully recapitulate the tumor tissue structure normally observed in humans include patient-derived xenografts, genetically engineered animals and environmentally induced models [31]. Another limitation is that we imaged the animals and analyzed the tissue at a single time point, i.e., 1 week post-RT. Experiments were designed in this way due to inherent limitations in terms of tumor growth rates, which are quite fast when using LLC and CT26 tumor cell lines. Measurements performed at different time points and at longer incubation periods would have provided a more accurate view of CAF dynamics post-RT. Nevertheless, despite these limitations, this study is one of the first to demonstrate radiation-induced effects on CAFs in vivo and to demonstrate the good performance of [^18^F]FAPI-74 in studying in vivo CAF dynamics in the context of therapy. Further work in more advanced and clinically relevant animal models is necessary to confirm the results presented in this study and to generate data that can be faithfully translated into clinical settings.

## Conclusions

This study provides compelling preclinical evidence supporting the use of [^18^F]AlF-FAPI-74 PET imaging as a noninvasive tool to monitor CAF dynamics in response to radiotherapy. Our findings demonstrate that CAFs constitute a relatively small population in subcutaneously transplanted tumor models, yet their abundance can be moderately increased following fractionated radiotherapy, particularly in the LLC/C57BL6 model. This radiation-induced increase in the number of FAP+ CAFs was consistently observed across multiple analytical platforms, including PET imaging, immunohistochemistry, and flow cytometry.

Importantly, [^18^F]AlF-FAPI-74 exhibited favorable radiochemical properties, high in vivo stability, and a biodistribution profile suitable for tumor imaging, with low background uptake in most organs and rapid renal clearance. The ability of the tracer to detect subtle changes in FAP expression postirradiation highlights its potential as a sensitive biomarker for assessing stromal responses to cancer therapy.

Despite the limitations inherent in subcutaneous tumor models, our results underscore the feasibility of using FAPI-based PET imaging to study therapy-induced stromal remodeling. These findings pave the way for future investigations in more physiologically relevant tumor models and clinical settings, where CAF-targeted imaging could inform treatment planning, monitor therapeutic efficacy, and potentially guide CAF-directed interventions.

## Electronic supplementary material

Below is the link to the electronic supplementary material.

Supplementary material 1

Supplementary material 2

Supplementary material 3

Supplementary material 4

## Data Availability

Datasets generated during and/or analyzed during the current study are available from the corresponding author on reasonable request.

## Abbreviations

BTV: Biological (active) tumor volume
CAFs: Cancer-associated fibroblasts
FAP: Fibroblast activation protein
FAPI: Fibroblast activation protein inhibitor
LLC: Lewis Lung Carcinoma
RT: Radiation therapy (or radiotherapy)
SUV: Standardized uptake values
TME: Tumor microenvironment
