# Supplementary figures and images for: Preclinical evaluation of FAP-targeted PET imaging to investigate CAF responses to radiotherapy
Source: Cancer Imaging. 2026 Feb 27;26:49. doi: 10.1186/s40644-026-01010-2 (PMC13049841; doi:10.1186/s40644-026-01010-2)

tumor

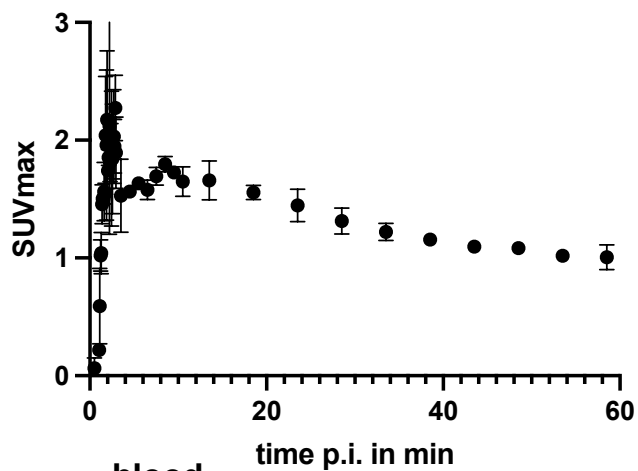

muscle

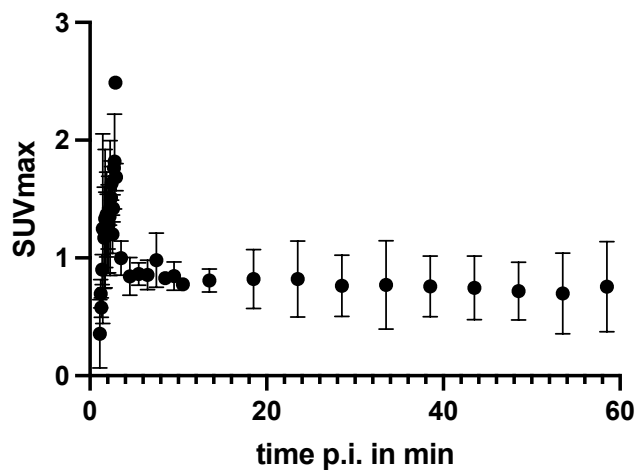

blood

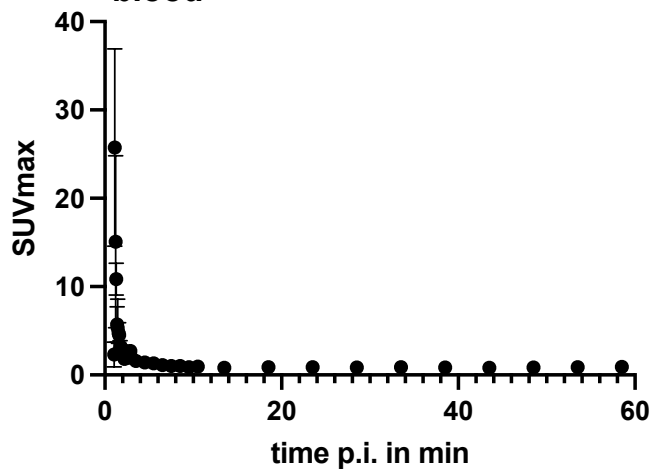

lungs

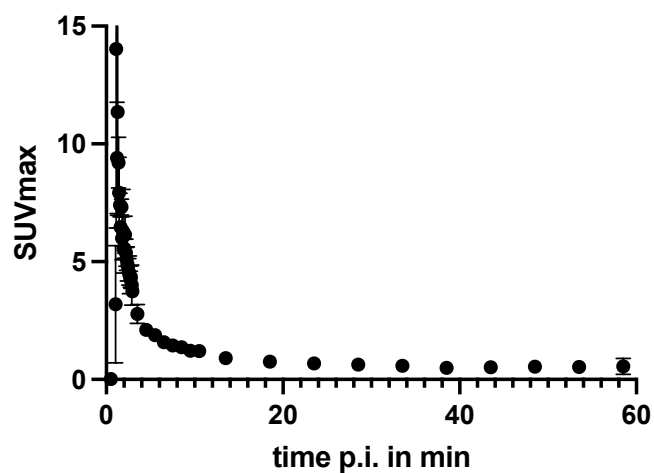

tumor-muscle-ratio

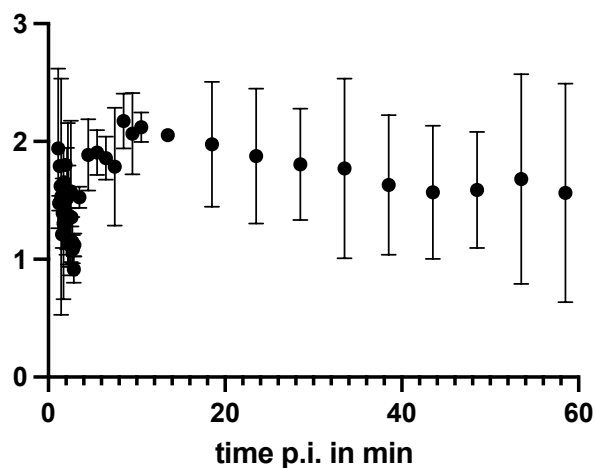

tumor-blood-ratio

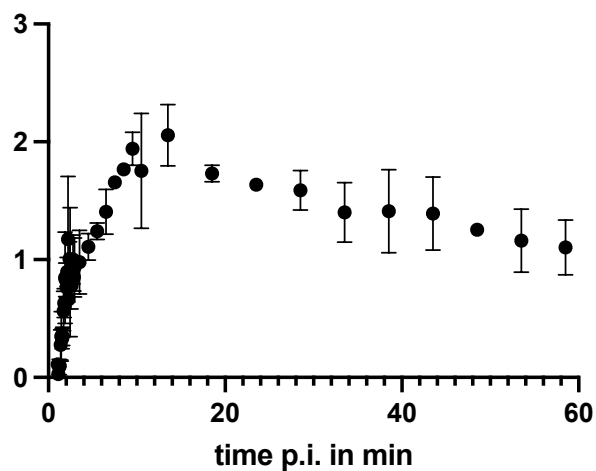

tumor-lung-ratio

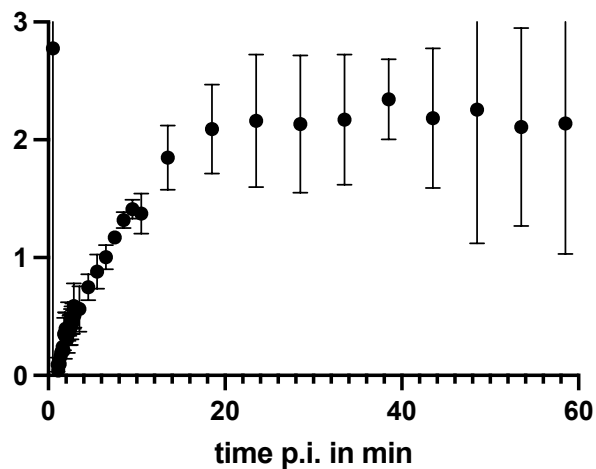

Supplement: Supplementary file 1 — Supplementary material 1 [file 40644_2026_1010_MOESM1_ESM.pdf]

tumor

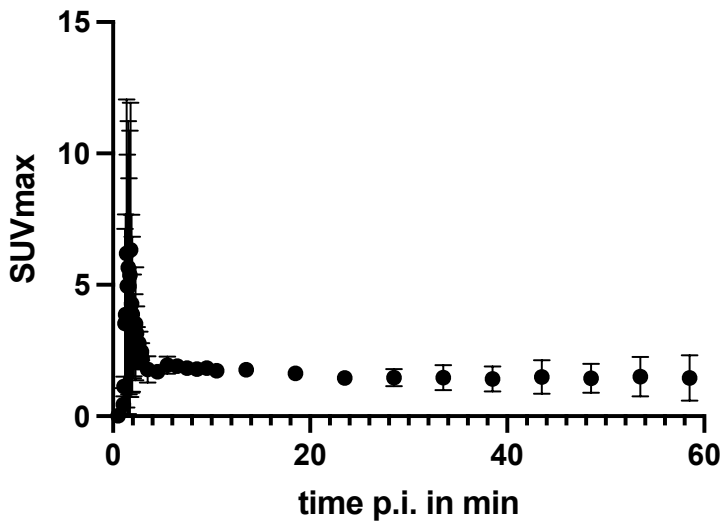

muscle

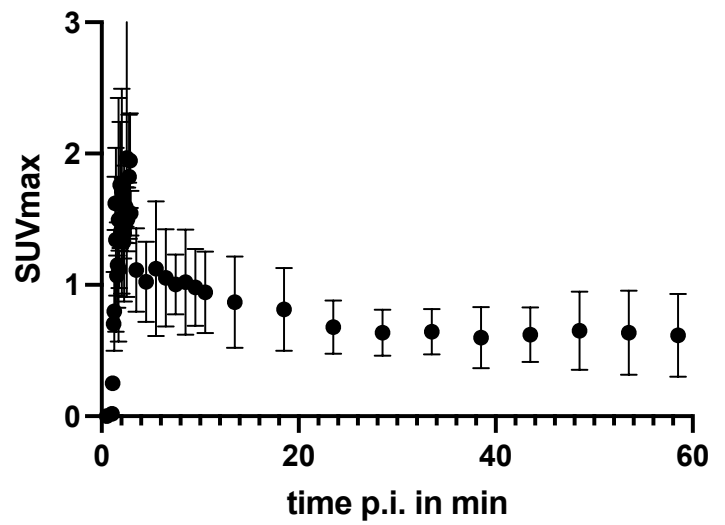

blood

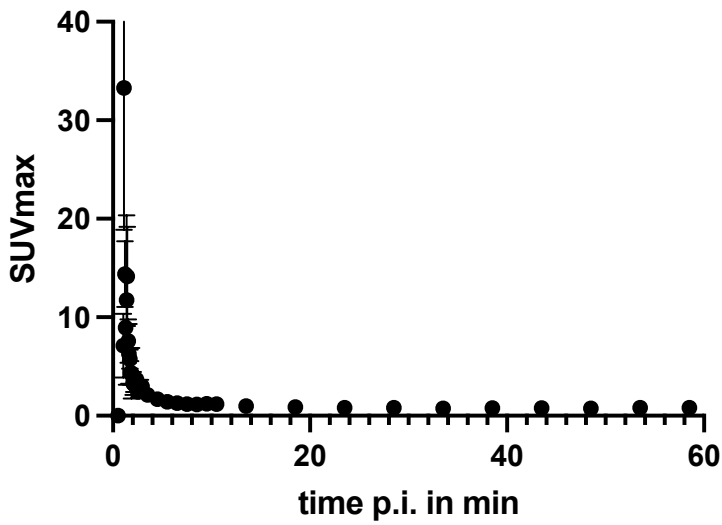

tumor-muscle-ratio

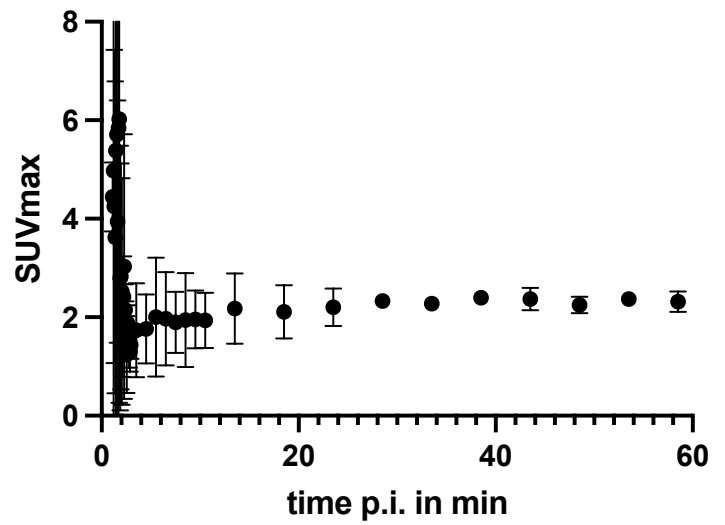

tumor-blood-ratio

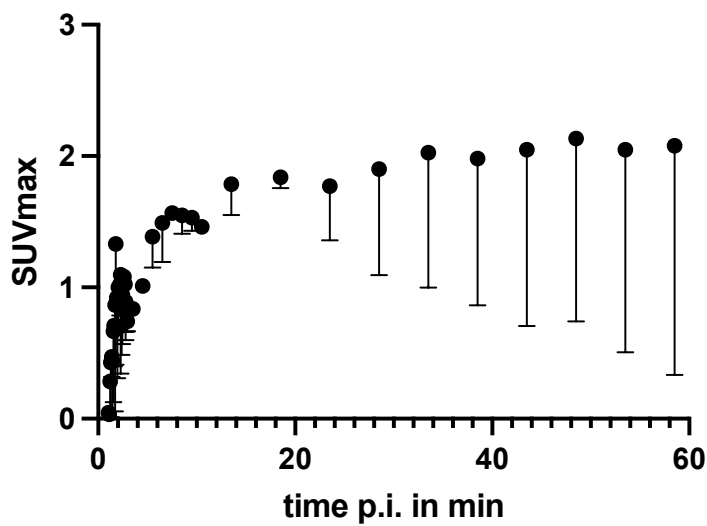

Supplement: Supplementary file 2 — Supplementary material 2 [file 40644_2026_1010_MOESM2_ESM.pdf]

## Slide 1
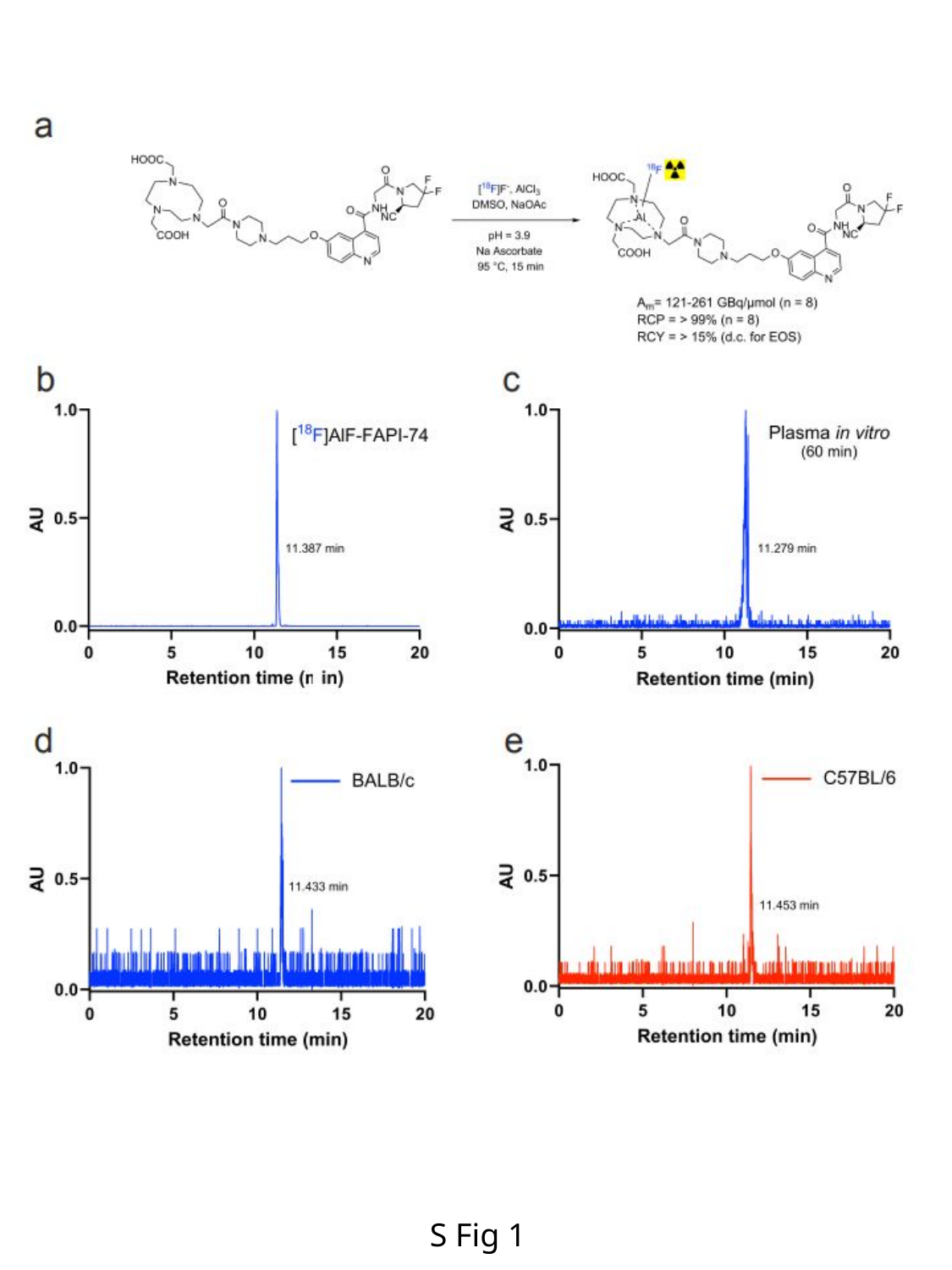

#
S Fig 1

Supplement: Supplementary file 3 — Supplementary material 3 [file 40644_2026_1010_MOESM3_ESM.pptx]

LL2 control

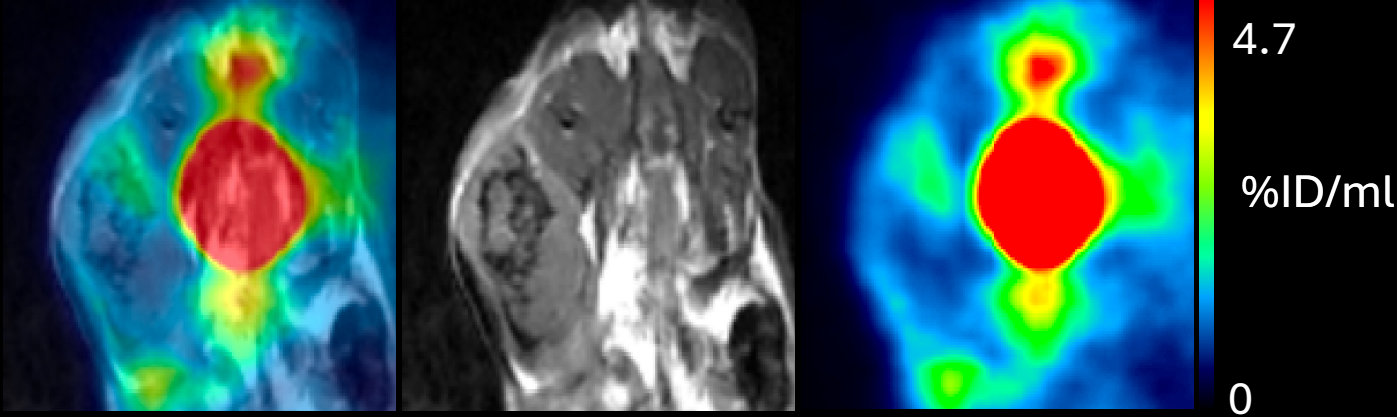

LL2 control

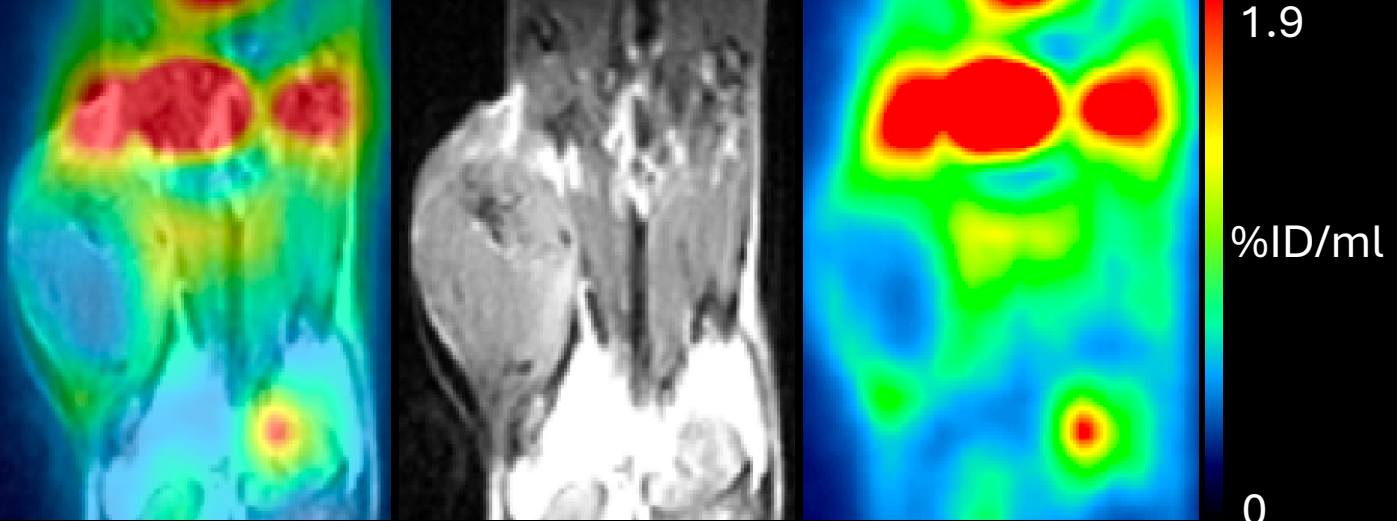

CT26 control

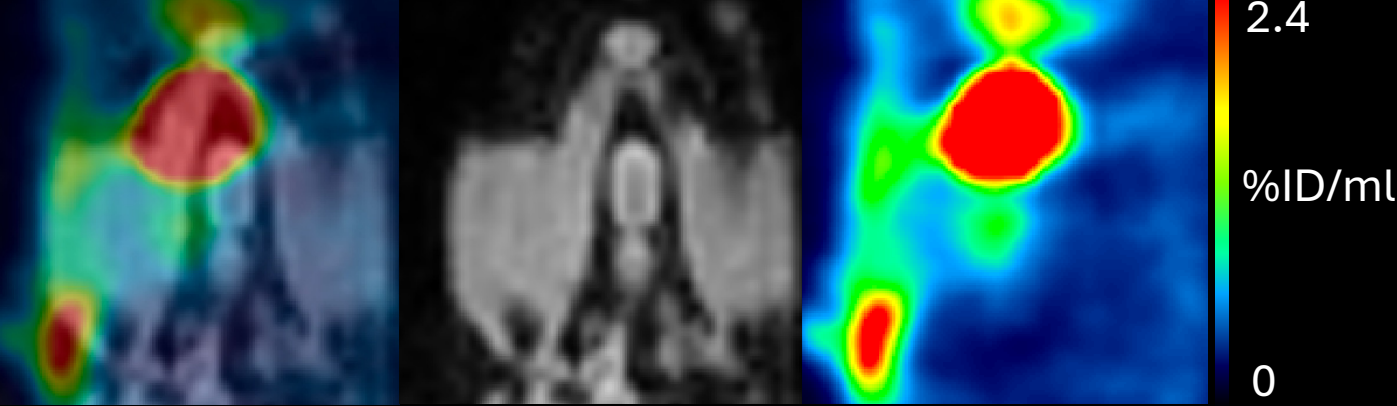

CT 26 control

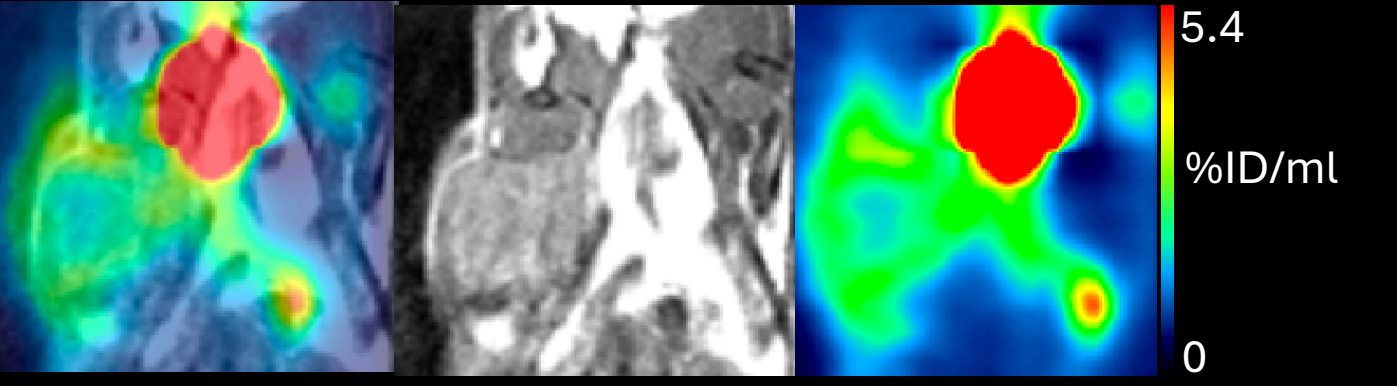

CT26 control

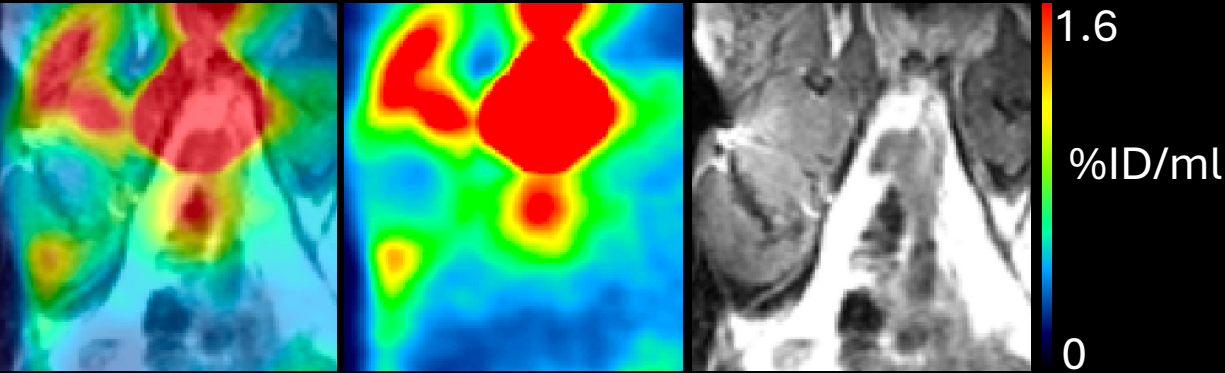

CT26 control

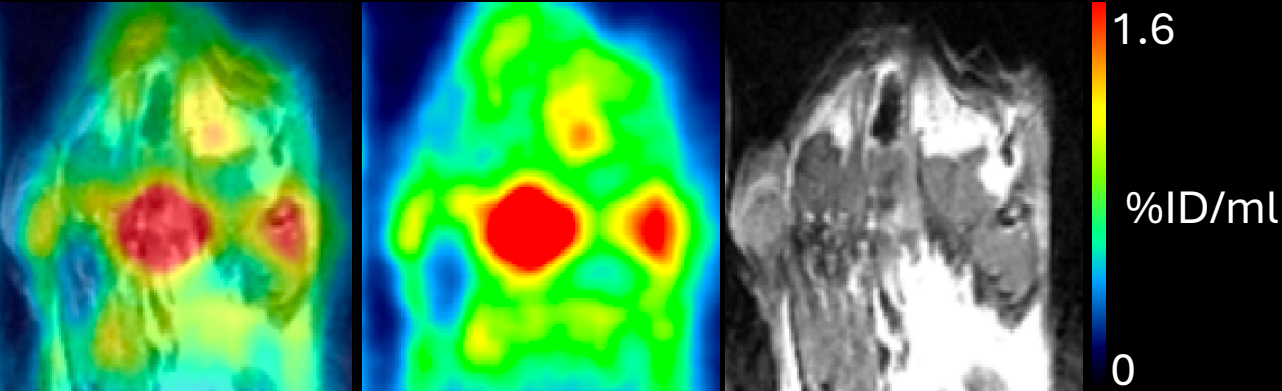

CT26 2x6Gy

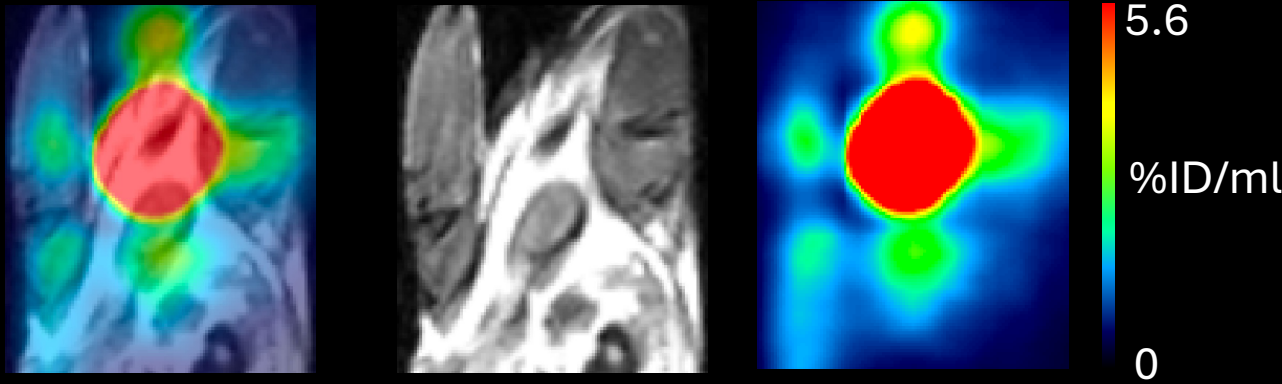

LL2 1x12Gy

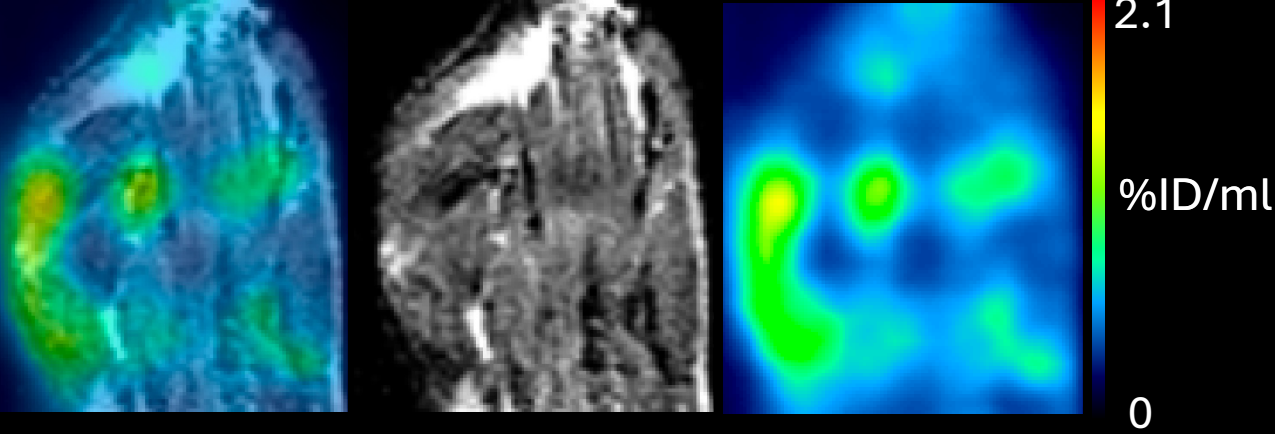

Supplement: Supplementary file 4 — Supplementary material 4 [file 40644_2026_1010_MOESM4_ESM.pdf]
